# Pepsin Hydrolysate from Surimi Industry-Related Olive Flounder Head Byproducts Attenuates LPS-Induced Inflammation and Oxidative Stress in RAW 264.7 Macrophages and In Vivo Zebrafish Model

**DOI:** 10.3390/md22010024

**Published:** 2023-12-28

**Authors:** H. H. A. C. K. Jayawardhana, N. M. Liyanage, D. P. Nagahawatta, Hyo-Geun Lee, You-Jin Jeon, Sang In Kang

**Affiliations:** 1 Department of Marine Life Science, Jeju National University, Jeju 63243, Republic of Korea; chathuri.k.j@stu.jejunu.ac.kr (H.H.A.C.K.J.); liyanagenm@jejunu.ac.kr (N.M.L.); pramuditha1992@jejunu.ac.kr (D.P.N.); hyogeunlee92@jejunu.ac.kr (H.-G.L.); 2 Seafood Research Center, Silla University, Busan 49277, Republic of Korea

**Keywords:** head byproducts, olive flounder, anti-inflammation, oxidative stress, LPS, macrophages, zebrafish: pepsin hydrolysate

## Abstract

Fish head byproducts derived from surimi processing contribute about 15% of the total body weight, which are beneficial to health because they contain essential nutrients. In this study, olive flounder (OF) was the target species in order to maximize the byproduct utilization. In RAW 264.7 macrophages, the seven hydrolysates from OF head byproducts were examined for their inhibitory potential against inflammation and the oxidative stress induced by lipopolysaccharides (LPS). The pepsin hydrolysate (OFH–PH) demonstrated strong anti-inflammatory activity via the down-regulation of NO production, with an IC50 value of 299.82 ± 4.18 µg/mL. We evaluated the inhibitory potential of pro-inflammatory cytokines and PGE2 to confirm these findings. Additionally, iNOS and COX-2 protein expressions were confirmed using western blotting. Furthermore, the results from the in vivo zebrafish model demonstrated that OFH–PH decreased the LPS-elevated heart rate, NO production, cell death, and intracellular ROS level, while increasing the survival percentage. Hence, the obtained results of this study serve as a platform for future research and provide insight into the mediation of inflammatory disorders. These results suggest that OFH–PH has the potential to be utilized as a nutraceutical and functional food ingredient.

## 1. Introduction

Consumers are experiencing increasing demand for fish and fish proteins, owing to their broad nutritional value and health benefits. Many different products with different market values can be obtained from fish. In addition to fish meat and other edible parts, the head, skin, and internal organs can be used as raw materials for producing value-added products [1]. The industrial processing of fish for human consumption generates more than 60% of the by-products, but the amount may depend on the raw materials, processing methods, and ultimate products required [2]. This accounts for approximately three-quarters of the total weight [3]. During fish processing, by-products such as skin and scales, intestinal parts (mainly the viscera, air bladder, and gonads), head parts, bones, and fins are generated [4].

As mentioned by Coppola et al. (2021), fish waste produced through industrial processing is directly discharged into oceans, which has become a serious environmental issue [5]. Hydrolysis is an effective process for overcoming this problem and can be used to recover underutilized fish proteins in more desirable and acceptable forms [6]. Enzymatic hydrolysis is an ideal and efficient method for obtaining bioactive peptides and lipids from fish discards [6,7]. However, there is one common characteristic when utilizing these enzymes to obtain protein hydrolysates: they should be categorized as food grade, and if they have a microbial origin, the microorganisms must not be pathogenic [8].

Fish protein hydrolysates are a potent source of protein supplements for human nutrition because of their balanced amino acid composition [2]. Protein hydrolysates obtained from aquatic resources provide evidence of promising bioactive agents with various bio-functional properties, including antioxidant, anti-inflammatory, antihypertensive, anticancer, and antibacterial properties [9]. Recently, several studies have been conducted on numerous bioactive peptides derived from food protein hydrolysates to assess their application in the biomedical sector. Simultaneously, some peptides have also gained attention as a functional dietary supplement [5,7,8].

South Korea is the world’s largest producer of olive flounder (*Paralichthys olivaceus*), and it is one of the major domestic industries. According to the reports of the Ministry of Ocean and Fisheries in South Korea, the olive flounder aquaculture production in 2020 was reported as 43,813 tonnes. Additionally, olive flounder accounts for 65% of marine-aquacultured fish consumption and 50% of fish production in South Korea [10]. Additionally, olive flounder has become one of the major species for surimi production in the last few years. During the surimi processing, nearly 50–70% solid waste is generated [11]. Moreover, approximately 14–20% is wasted at the fish heading step in surimi production plants [5]. A considerable proportion is acquired by the head when compared to the body size of the fish. This study mainly focused on the generation of surimi industry-related byproducts to maximize the utilization of underutilized fish proteins. Therefore, enzymatic hydrolysates obtained from the head byproducts of olive flounder were studied against LPS-induced oxidative stress and inflammation-associated functional responses. Seven commercial proteases (Alcalase, Flavourzyme, Kojizyme, Neutrase, Protamax, pepsin, and trypsin) were used to obtain fish protein hydrolysates and in vitro and in vivo experiments were conducted to evaluate their bioactivities.

## 2. Results

### 2.1. Yield and Chemical Composition

Table 1 lists the proximate compositions of the dried OFH samples. According to these data, protein is the most abundant component, accounting for approximately 56% of the raw material. Both the lipid and ash contents were 17%, which is considerably higher than the whole fish [12].

Table 2 listed the yield and the protein content of the seven different hydrolysates. According to the data, the extraction yield ranged from 26.67 ± 1.25–46.50 ± 0.50%, while pepsin hydrolysate possessed the highest extraction efficiency. Additionally, when comparing the protein content of the hydrolysates, protamax hydrolysate showed the highest protein content at 52.06 ± 0.15%.

### 2.2. Cytotoxicity, NO, and Intracellular ROS Production in RAW 264.7 Cells

**Figure 1:**
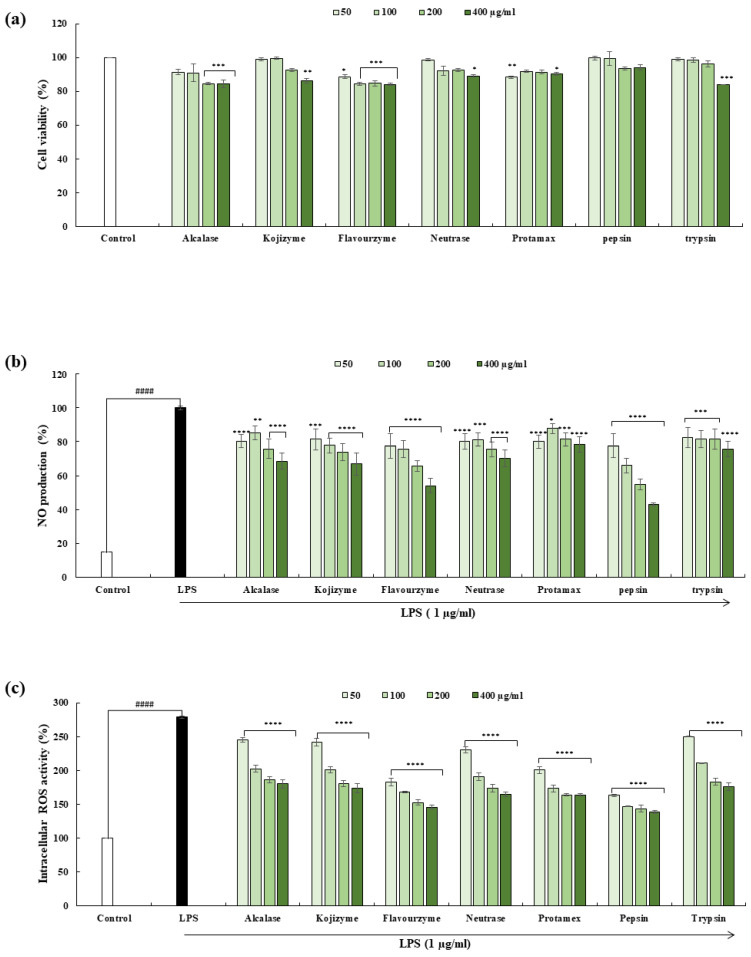
In vitro anti-inflammatory activities of OFH hydrolysates in Raw 264.7 cells. Cytotoxicity of enzyme-assistant extracts of OFH in Raw 264.7 cells (**a**), inhibitory effects of enzymatic hydrolysates of OFH against LPS-induced NO production in Raw 264.7 cells (**b**), and intracellular ROS scavenging activity of enzymatic hydrolysates of OF head byproducts against LPS-induced Raw 264.7 cells (**c**). All the experiments were tested in duplicates and represented as the mean ± SE. Significance was compared relative to the LPS-treated group at * *p* < 0.05, ** *p* < 0.01, *** *p* < 0.005, **** *p* < 0.0001, and ^####^ *p* < 0.0001 relative to the control.

To determine the anti-inflammatory potential of the seven different hydrolysates, first we examined their cytotoxicity in RAW 264.7 macrophages. Most of the hydrolysates exhibited cell toxicity at the highest concentration. However, OFH–PH did not show any significant alterations in viability or cell morphology at various concentrations (50–400 µg/mL), as shown in Figure 1a. In order to determine whether it regulates the production of inflammatory mediators, we specifically measured its effect on the ROS and NO levels in the LPS-stimulated RAW 264.7 macrophages. Most of the hydrolysates showed a dose-dependent inhibition of NO production in response to LPS (Figure 1b). Table 3 demonstrates the obtained IC_50_ values. However, OFH–PH possesses the best activity among the seven different hydrolysates, with an IC_50_ value of 299.82 ± 4.18 µg/mL. All of the hydrolysates effectively reduced the intracellular ROS production, while the OFH–PH treatment showed the best effect (Figure 1c). These results indicated that OFH–PH potently inhibited LPS-induced inflammation in RAW 264.7 macrophages by reducing NO and ROS production.

### 2.3. Amino Acid Composition Analysis and Molecular Weight Determination

The amino acid composition of OFH–PH is summarized in Table 4. In OFH–PH, the most abundant amino acids were found to be glycine (15.59%) and Glutamic acid (12.89%), followed by alanine (8.58%), proline (8.51%), arginine (8.30%), and aspartic acid (8.16%). The total percentage of essential amino acids was 85.7%, which is comparatively high, therefore indicating that OFH–PH can be used as a good source of essential amino acids.

**Figure 2:**
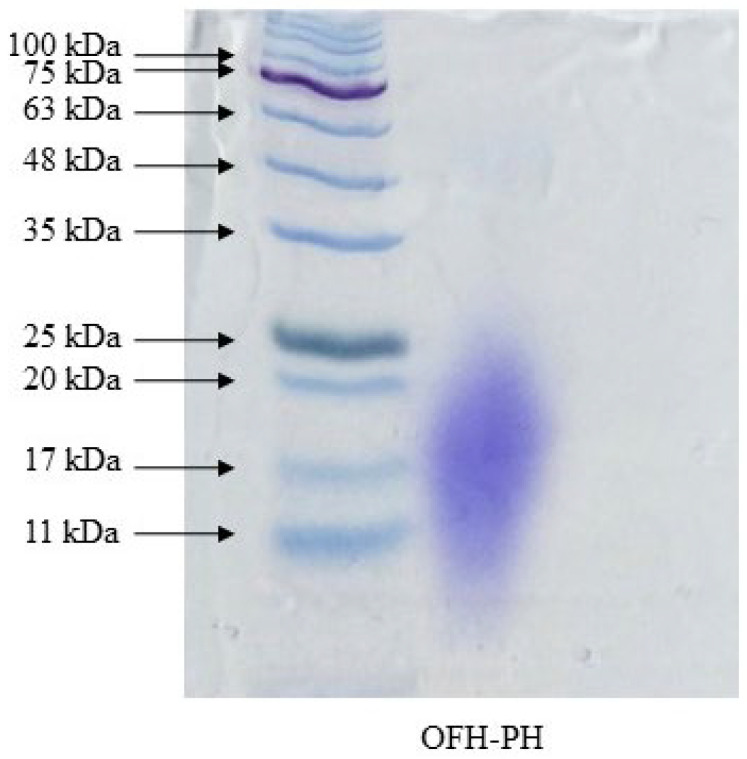
The molecular weight distribution of OFH–PH. The SDS-PAGE results indicate the molecular weight distribution of OFH–PH stained by Coomassie blue staining.

The characterization of the molecular weights of OFH–PH through SDS-PAGE (Figure 2) showed the presence of strange bands below the molecular weight of 25 kDa, which indicated that pepsin enzyme was able to produce small-sized peptides. Previous studies have shown that low molecular weight hydrolysates have comparatively high bioactive properties. Bhaskar et al. (2008) stated that fish protein hydrolysates with a high nutritional value are rich in low molecular weight peptides [13], and the successful production of such desired peptides from OFH indicates its potential for use in functional food products.

### 2.4. PGE2, and Pro-Inflammatory Cytokine Production

**Figure 3:**
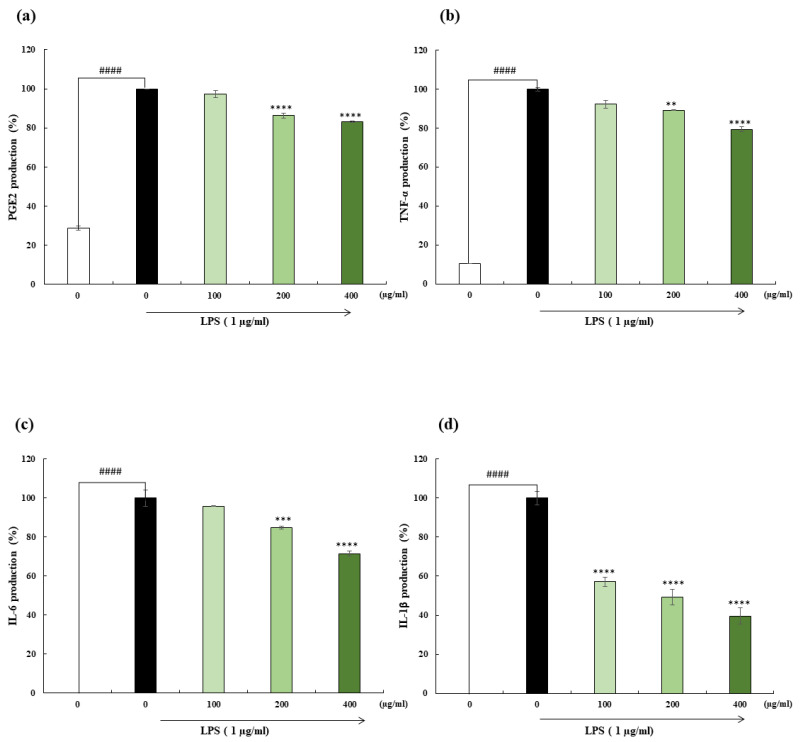
Effect of OFH–PH on LPS-induced pro-inflammatory cytokine, and prostaglandin E2 (PGE2) expression. PGE2 (**a**), tumor necrosis factor (TNF)-ɑ (**b**), interleukin (IL)-6 (**c**), and IL-1β (**d**), expression levels. All the experiments were tested in duplicates and represented as the mean ± SE. Significance was compared relative to the LPS-treated group at ** *p* < 0.01, *** *p* < 0.005, **** *p* < 0.0001, and ^####^ *p* < 0.0001 relative to the control.

PGE2 is a key mediator which regulates inflammatory responses. The increased production of pro-inflammatory cytokines, including TNF-α, IL-6, and IL-1β, plays a major role in chronic inflammation [14]. As illustrated in Figure 3, PGE2 and pro-inflammatory cytokines production was examined after the treatment with OFH–PH. LPS stimulation increased the production of PGE2 and pro-inflammatory cytokines compared to the control group. However, in the sample treatment, the enhanced levels were decreased dose-dependently. The PGE2 levels decreased slightly to 83.10% at the highest concentration. Additionally, at the highest concentration, TNF-α, IL-6, and IL-1β decreased by up to 79.33%, 71.53%, and 39.63%, respectively.

### 2.5. iNOS and COX-2 Expression

**Figure 4:**
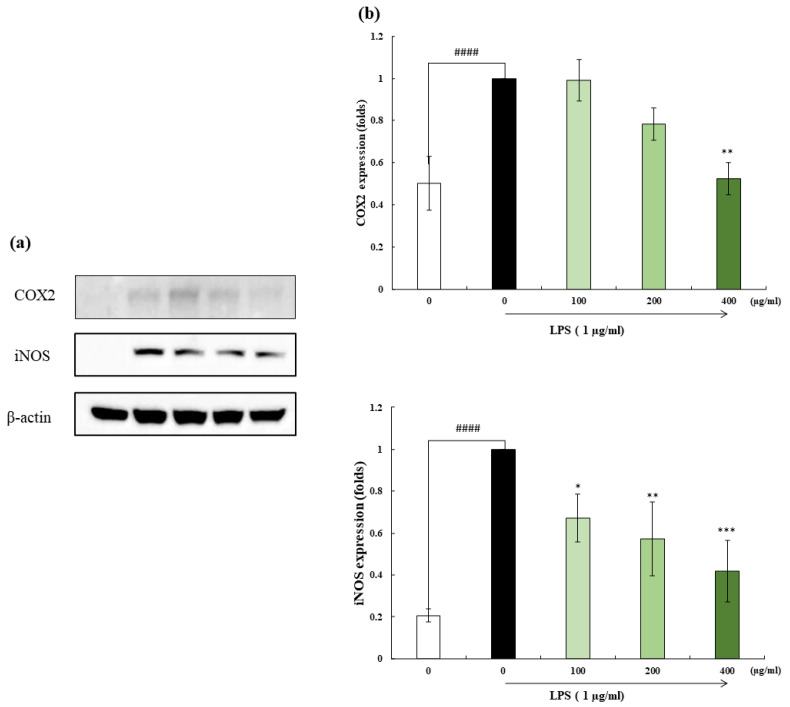
Effect of OFH–PH on LPS-induced iNOS, and COX-2 expression. Representative images of phosphorylation levels of iNOS and COX-2 (**a**), proteins were determined using western blot. Statistical representation (**b**). The western blot bands were visualized with the FUSION SOLO Vilber Lourmat system and quantified the intensity using the ImageJ (version 1.4) software. All the experiments were tested in duplicates and represented as the mean ± SE. Significance was compared relative to the LPS-treated group at * *p* < 0.05, ** *p* < 0.01, *** *p* < 0.005, and ^####^ *p* < 0.0001 relative to the control.

To determine the inhibitory activity on the iNOS and COX-2 expression levels, western blotting was performed. iNOS and COX-2 play significant roles in mediating immune responses in cells [15]. The results indicated that the LPS-stimulated expression levels of the above-mentioned proteins were comparable to those in the control cells. Pre-treatment with OFH–PH down-regulated the expression levels of iNOS in LPS-stimulated cells dose-dependently (Figure 4). However, only the highest concentration of the sample showed a significant reduction in COX-2 expression. Hence, these results evidenced that the inhibitory effect of OFH–PH against LPS-induced inflammation was strongly regulated by NO downregulation.

### 2.6. In Vivo Studies

**Figure 5:**
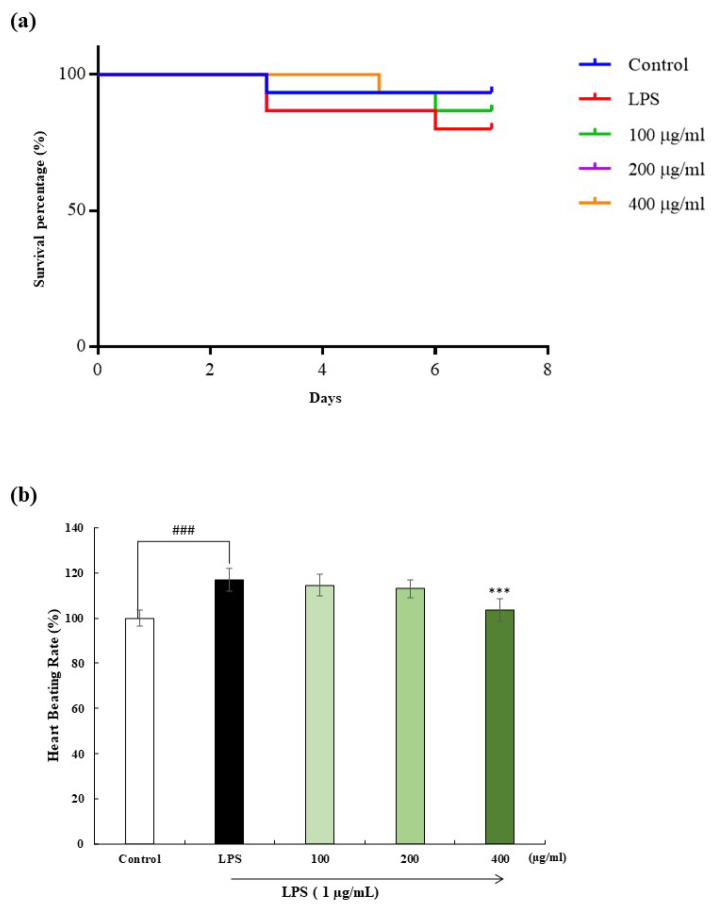
Effect of OFH–PH on LPS-induced alterations in survival rate and heartbeat rate of zebrafish model. LPS-induced survival rate (**a**), and heartbeat rate (**b**). Data are represented as the mean ± SE. Significance was compared relative to the LPS-treated group at *** *p* < 0.005, and ^###^ *p* < 0.005relative to the control.

The survival percentage and heartbeat rate were monitored to determine the effect of OFH–PH on LPS-induced zebrafish embryos. As demonstrated in Figure 5a, a survival rate of around 70% was observed in the LPS-stimulated embryos. However, the reduced survival rate was significantly increased dose-dependently with the treatment of OFH–PH. Furthermore, Figure 5b illustrates that LPS slightly increased the heart rate, whereas OFH–PH normalized the elevated heartbeat rate of the LPS-induced zebrafish embryos. Inflammatory responses and oxidative stress stimulate cell death, the overproduction of ROS, as well as NO production; therefore, the protective effect of OFH–PH against LPS-induced cell death, intracellular ROS activity, and NO production in zebrafish embryos was evaluated.

**Figure 6:**
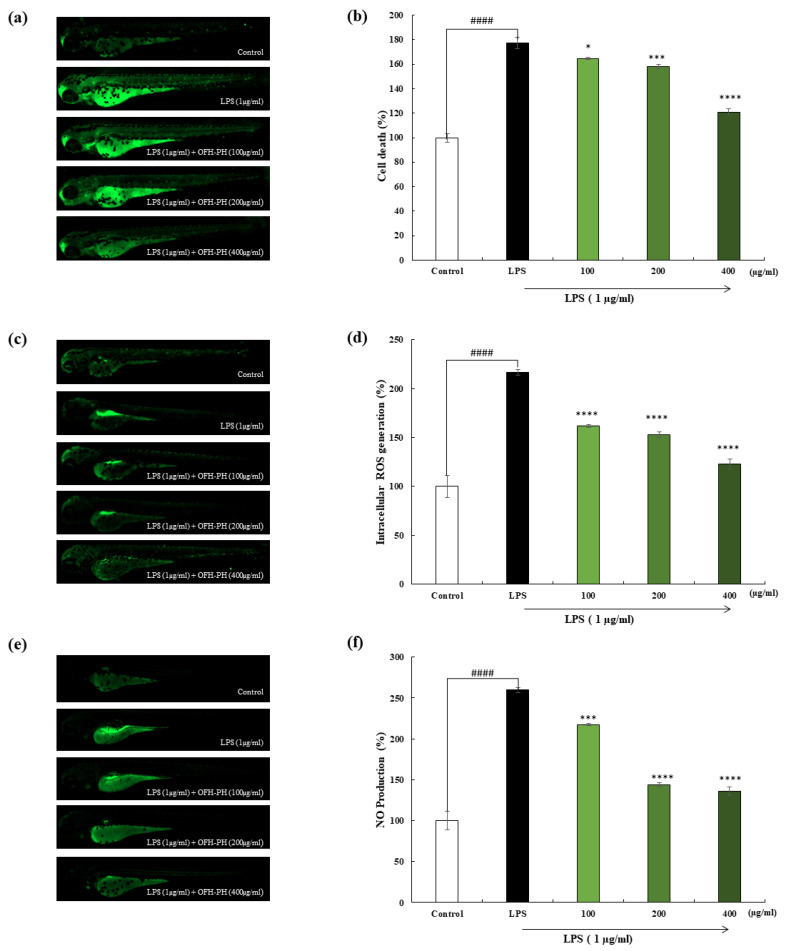
Effect of OFH–PH on LPS-induced damage in the zebrafish model. LPS-induced cell death, stained with AO (**a**), LPS-induced ROS activity, stained with DCFH-DA (**c**), LPS-induced NO production, stained with DAF-FM-DA (**e**) and captured under fluorescent microscope. Quantitative analysis was conducted using ImageJ (version 1.4) software and graphically represented in (**b**,**d**,**f**), respectively Significance was compared relative to the LPS-treated group at * *p* < 0.05, *** *p* < 0.005, **** *p* < 0.0001, and ^####^ *p* < 0.0001 relative to the control.

As shown in Figure 6a,b, the LPS-induced cell death of the zebrafish embryos was confirmed by measuring the acridine orange and fluorescent intensity. In comparison to the control group, LPS-induced cell death was found to be 180%. The OFH–PH treatment led to a reduction in cell death by up to 120% at 400 µg/mL. As illustrated in Figure 6c,d, the ROS production in the LPS-treated zebrafish embryos increased by up to around 220% compared to the control group. However, significantly reduced levels of ROS production—of 160%, 150%, and 120%—was observed in the zebrafish embryos exposed to LPS and OFH–PH at different concentrations of 100, 200, and 400 g mL, respectively. In addition, the LPS-induced NO production was evaluated using DAF-FM-DA fluorescent dye. As demonstrated in Figure 6e,f, the LPS-induced group revealed 260% NO production, while the OFH–PH-treated groups showed a significant reduction in a dose-dependent manner. The highest concentration of OFH–PH exhibited reduced NO production of up to 125%. These results demonstrated that the OFH–PH treatment significantly decreased the NO production. Therefore, the OFH–PH treatment dose-dependently led to a dramatic reduction in cell death, intracellular ROS production, and NO production. The obtained results demonstrate that OFH–PH has significant anti-inflammatory potential.

## 3. Discussion

In this study, we used OFH surimi byproducts, which generated a considerable amount (14–20%) compared to the whole fish. As fish proteins are superior to plant-derived proteins, as well as being composed of a better amino acid balance compared to other animal proteins, fish by-products can be utilized in many novel applications. In addition to containing essential nutrients, protein hydrolysates have balanced amino acid profiles. Due to the short-chain peptides and free amino acids produced by hydrolysis, these products can be used in the nutraceutical and functional food industries [11].

The worldwide production of protein hydrolysates is enormous. Chemical and biological methods are the most common methods for manufacturing protein hydrolysates in the industry. The chemical methods include acidic and alkaline hydrolysis. As chemical methods are relatively inexpensive and easy to perform, they are preferred for producing protein hydrolysates on an industrial scale. However, chemical hydrolysis results in the cleavage of nonspecific peptide bonds, leading to a heterogeneous yield of peptides and reduced nutritional quality of the products [11]. Therefore, in this study, we used enzymatic hydrolysis to obtain pepsin hydrolysate (OFH–PH) and evaluated its anti-inflammatory potential.

Under normal circumstances, inflammation is a physiological response to pathogens, injuries, or other stimuli that enable the host to defend itself; however, the prolonged overproduction of inflammatory mediators (e.g., pro-inflammatory cytokines) can contribute to numerous diseases, either directly or indirectly [16]. To expose the effects of OFH–PH on the inflammatory factors, mediators, and related proteins, RAW 264.7 macrophages were used as the research object in this study. Numerous inflammatory responses are triggered by activated RAW 264.7 macrophages. In the context of host defense mechanisms, inflammation plays an important role. LPS is a bacterial endotoxin which acts as a powerful inflammatory signal that increases the expression of pro-inflammatory cytokines (TNF-, IL-6, and PGE2), NO, iNOS, and COX-2, as well as different signaling pathways in monocytes and macrophages, thereby mediating inflammatory responses [17,18]. Thus, the inhibition of inflammatory mediators can be an effective way to regulate inflammation. Chemically based medicines such as indomethacin, prednisone, ibuprofen, and naproxen are most commonly used for the treatment of inflammation. However, these drugs have side effects, including kidney damage [19]. Therefore, scientific studies are currently focusing on naturally derived drugs because consumers are more aware of their hazardous health effects.

The expression of COX-2 and iNOS proteins, which stimulate the production of PGE2 and NO, respectively, can be activated by LPS or other cytokines [20]. NO plays a key role in pathogenesis and has diverse physiological and pathological functions [21]. When macrophages are activated, they release high concentrations of NO as a result of iNOS expression [15]. During inflammation, COX-2 regulates PGE2 synthesis via the arachidonic acid pathway [22]. The treatment with OFH–PH significantly downregulated the production of NO, PGE2, COX-2, and iNOS in the LPS-stimulated RAW 267.4 cells in a dose-dependent manner. These results were confirmed using western blotting. Several studies have confirmed the anti-inflammatory potential of the hydrolysates obtained from various fish species. Similar results were obtained in a study on the anti-inflammatory effects of pepsin, trypsin, and chymotrypsin hydrolysates in sweetfish on LPS-induced RAW 264.7 cells [23].

NO plays a crucial role in the incidence and progression of inflammation by altering the permeability of the cell membrane, which encourages the production of inflammatory molecules such as TNF-α, IL-6, IL-1β, and PGE2 [24]. LPS promotes the synthesis and release of pro-inflammatory cytokines and PGE2, aggravating the inflammatory response. The present study demonstrates that OFH–PH significantly downregulates the production of these mediators. Inflammation encourages the generation of ROS, and this excessive production of ROS results in lipid peroxidation, which can severely damage cells; increased ROS levels are frequently identified as key signs of injury [25]. The results evidenced that OFH–PH dose-dependently inhibited LPS-induced intracellular ROS generation.

In the research field, different in vivo animal models are commonly used for further confirmation of biological activities. In view of its rapidity and sensitivity, the zebrafish embryo model has become a commonly used animal model for the investigation of anti-inflammatory drug development [26]. Additionally, the zebrafish model shows similarities with embryogenesis in higher vertebrates [27]. This study investigated the inflammation and oxidative stress inhibitory potential of OFH–PH on zebrafish embryos after LPS exposure. In general, DA fluorescent probes including Acridine orange (AO), DAF-FM-DA, and DCFH-DA are used to evaluate inflammatory and oxidative stress-related physiochemical alterations [11]. AO is a metachromatic dye used for selective stains of DNA and RNA through intercalation or electrostatic attraction in living embryos. AO preferentially stained necrotic or very late apoptotic cells with damaged plasma membrane permeability [28]. DCFH-DA is an oxidation-sensitive fluorescent probe dye that is enzymatically hydrolyzed intracellularly to non-fluorescent DCFH through non-specific esterases. Due to the presence of cellular ROS, DCFH can be further oxidized to dichlorofluorescein (DCF), which is a highly fluorescent compound. The obtained results revealed that OFH–PH has the potential to regulate inflammatory responses in in vivo zebrafish model. Similarly, previous studies also confirmed that fish hydrolysates are potential sources of anti-inflammatory agents [11].

These results demonstrate that pepsin OFH–PH has great potential as an anti-inflammatory candidate in the production of functional foods. These results demonstrate that OFH–PH can down-regulate the expression of NO, pro-inflammatory cytokines, and iNOS; in vivo studies further confirmed the findings. Collectively, the results concluded that OFH–PH has the potential as an anti-inflammatory agent through the downregulation of NO production and protein expression. In addition, the study showed that OFH–PH obtained from surimi byproducts is a good source of natural anti-inflammatory agents and can also be utilized as an ingredient in food formulations or as a functional food.

## 4. Materials and Methods

### 4.1. Chemicals

The murine macrophage cell line (RAW 264.7) was purchased from the Korean Cell Line Bank (Seoul, Republic of Korea). Dulbecco’s modified Eagle’s medium (DMEM), penicillin-streptomycin, and fetal bovine serum (FBS) were purchased from Welgene Inc., Daegu, Republic of Korea. Dimethyl sulfoxide (DMSO) and 3-[4,5-dimethylthiazole-2-yl]-2,5-diphenyltetrazolium bromide (MTT) were purchased from Sigma Chemical Co. (St. Louis, MO, USA). Primary antibodies against iNOS, COX-2, and secondary antibodies were purchased from Cell Signaling Technology (Beverly, MA, USA). BCA protein Assay Kit and Enhanced chemiluminescence reagent were purchased from Thermo Fisher Scientific (Waltham, MA, USA). PGE2 and pro-inflammatory cytokines, including TNF-α, IL1-β, and IL-6 ELISA kits were purchased from R&D Systems Inc., Minneapolis, MN, USA. Proteases [Alcalase (2.5 AU-A/g), Kojizyme (800 LAPU/g), Neutrase (0.8 AU-N/g), Flavourzyme (500 LAPU/g), Protamex (1.5 AU-A/g), trypsin (800 USP/mg), and pepsin (1:3000 or 3000 units/mg)] were purchased from Novozymes Ltd. (Bagsvaerd, Denmark) and Daejung Chemicals and Metals Co., Ltd., Busan, Republic of Korea. acridine orange (AO), DAF-FM-DA, and DCFH-DA staining were obtained from Thermo Fisher Scientific (Waltham, MA, USA) and Sigma Chemical Co. (St. Louis, MO, USA). All chemicals and reagents employed in the different analyses were of analytical grade and are commercially available.

### 4.2. Fish Byproduct Materials

Olive flounder (*Paralichthys olivaceus*) surimi processing discards (Jeju Tamna Seafood Co., Ltd., Jeju, Republic of Korea) were used as substrates for enzymatic hydrolysis to obtain the fish hydrolysate. The muscles of the fish were separated for processing, and the remaining discards were manually removed. Samples (olive flounder heads, OFH) were preserved under frozen conditions from the processing plant to the laboratory. The samples were washed thoroughly with fresh water to remove all debris, including salts, and were immediately freeze-dried. Dried samples were subsequently homogenized through grinding and stored at −20 °C until used for hydrolysis [11].

### 4.3. Preparation of Samples: Enzyme Hydrolysis

OFH was hydrolyzed with 7 different enzymes under optimal conditions. The enzyme hydrolysis reactions were performed at the following optimum reactive conditions for each enzyme: Alcalase (50 °C, pH 8.0), Protamex (40 °C, pH 6.0), Kojizyme (40 °C, pH 6.0), Flavourzyme (50 °C, pH 7.0), Neutrase (50 °C, pH 6.0), pepsin (37 °C, pH 2.0), and trypsin (37 °C, pH 7.6). Before use, the samples were ground to a powder and homogenized using a mincer. For one gram of the sample, 100 mL of distilled water was added and the pH was adjusted to the optimum value before digestion. The enzyme was then added at an enzyme/substrate ratio of 1:100 (*v*/*w*), and hydrolytic reactions were performed under the optimum reaction conditions for 24 h in a shaker. To inactivate the enzyme, the digests were boiled for 10 min at 100 °C. Using a centrifuge, the samples were centrifuged to remove the residues under the conditions of 3000 rpm for 20 min at 4 °C. Then, the pH of the samples was adjusted to 7, lyophilized, and stored at −20 °C until used for further experiments [11].

### 4.4. Proximate Composition of the Samples

The chemical compositions (protein, polysaccharide, lipid, ash, and moisture) of the freeze-dried samples were measured according to the Association of Official Analytical Chemists (AOAC) methods. The Soxhlet method, Kjeldahl method, and phenol-sulfuric acid reaction were used to determine the crude lipid, protein, and polysaccharide contents, respectively. The ash content in the sample was analyzed through calcination at a temperature of 550 °C for 6 h in a furnace, while the moisture content was determined by keeping the sample in a dry oven at 105 °C for 24 h [29].

### 4.5. Cell Culture

#### 4.5.1. Raw 264.7 Macrophage Cell Line

The Raw 264.7 macrophages were cultured in Dulbecco’s Modified Eagle’s medium (DMEM) supplemented with 10% fetal bovine serum albumin (FBS) and 1% antibiotics (penicillin and streptomycin). The cells were maintained at a controlled temperature (37 °C) and 5% CO_2_ (Sanyo MCO-18AIC; Moriguchi, Japan) [30].

#### 4.5.2. Sample Toxicity

The cell viability was analyzed using an MTT assay. This experiment was conducted according to a previously described method. Briefly, macrophages were seeded at a seeding rate of 1 × 10^5^ cells/well in 96 well plates and incubated for 24 h, followed by treatment with different concentrations (50–400 µg/mL). Following 24 h of incubation, 50 µL MTT solution was added to the cells for a period of 2–3 h. After removing the MTT solution, 200 µL of DMSO reagent was added to dissolve stained formazan crystals and the absorbance was measured at 540 nm measured using a SpectraMax M2 microplate reader (Molecular Devices, San Jose, CA, USA) [30].

#### 4.5.3. LPS-Induced NO Production

To evaluate the effects of hydrolysates, LPS-induced RAW 264.7 macrophages were tested for NO levels using the Griess method. Various concentrations of samples (50–400 µg/mL) were pretreated for 1 h on the cells (1 × 10^5^ cells/well) and then incubated with LPS (1 µg/mL) for 24 h. An equal volume of the culture supernatant was mixed with Griess reagent (1% sulfanilamide and 0.1% N-[naphthyl] ethylenediamine dihydrochloride in 2.5% H_3_PO_4_) for 10 min at room temperature. The absorbance was measured at 540 nm using a microplate reader [30].

#### 4.5.4. LPS-Induced Intracellular ROS Production

A DCF-DA assay was performed to evaluate the effect of hydrolysates on intracellular ROS production. Before stimulation with LPS (1 µg/mL), the cells were pretreated with different doses of 50–400 µg/mL. After incubation, DCFH-DA reagent (500 µg/mL) was added for 1 h, and the fluorescence was measured using a microplate reader at 485 nm for excitation and 525 nm for emission [11].

### 4.6. Amino Acid Composition

The amino acid composition of OFH–PH was analyzed using S433-H, Sykam GmbH amino acid analyzer (Germany). Amino acids were examined using a cation separation column (LCA K06/Na, 4.6 × 150 mm) with buffer flow rates of 0.45 mL/min and reagent flow rates of 0.25 mL/min at wavelengths of 440 and 570 nm using fluorescence spectrophotometer [31].

### 4.7. Molecular Weight

The characterization of OFH–PH based on its molecular weight was performed using 12% Sodium dodecyl sulfate-polyacrylamide gel electrophoresis (SDS-PAGE). Samples were heated at 100 °C for 3 min before starting the electrophoresis. Then, the gel was stained with Bio-Rad Coomassie Blue R-250 followed by destaining several times. The bands obtained from the samples were compared with reference to the migration of the wide range molecular weight standard [32].

### 4.8. Pro-Inflammatory Cytokine and PGE2 Production

264.7 macrophages were treated with OFH–PH, followed by LPS (1 µg/mL), for 1 h. After 24 h incubation, the culture media were separated and collected. The collected supernatant was used to determine the expression levels of PGE2 and pro-inflammatory cytokines (TNF-α, IL-6, and IL-1β) using ELISA [30]. The experiments were conducted according to the manufacturer’s instructions.

### 4.9. Western Blot Analysis

Western blot analysis was performed according to a previously described method to determine the protein expression levels. Three different concentrations (100–400 µg/mL) were selected and treated to the cells following a procedure similar to the above-mentioned procedure. Briefly, the cells were lysed and centrifuged to collect the supernatant (15,000 rpm at 4 °C). The protein concentration of each supernatant was determined using a BCA protein assay kit. Proteins were separated using 12% sodium dodecyl sulfate-polyacrylamide gel electrophoresis. The separated protein bands were transferred to a nitrocellulose membrane and blocked with 5% skim milk. The membranes were incubated with primary and secondary antibodies, followed by visualization of the protein bands [33]. Signals were developed using a chemiluminescent substrate and bands were visualized using a FUSION SOLO Vilber Lourmat system. The results were quantified using the ImageJ software. Using western blotting, iNOS and COX-2 proteins were examined.

### 4.10. Survival Percentage, and Heatbeating Rate of Zebrafish Model

Zebrafish embryos obtained through natural spawning induced by light were collected within 30 min. After 7–9 h post-fertilization (hpf), the collected embryos were transferred to a 12-well plate (15 per well) and 1 mL of embryo media was added. Different concentrations of OFH–PH (100, 200, and 400 μg/mL) were treated and incubated for 1 h. Following the incubation, the embryos were induced by LPS (1 µg/mL) and continued the incubation for 24 hpf. Live embryos were counted 3 d after fertilization in order to determine the survival rate. For further analysis, surviving larvae were used. As stated in prior studies, the heart rate of the atrium and ventricles was recorded for 1 min after 2 dpf with a microscope [26].

### 4.11. Cell Death, NO Production, and Intracellular ROS Activity in Zebrafish Model

Based on a previous study, cell death, NO production, and intracellular ROS levels were measured using acridine orange (AO), DAF-FM-DA, and DCFH-DA staining, respectively. Following staining, larvae from the zebrafish were rinsed twice using embryo culture media to remove excess stain and anesthetized with 2-phenoxyethanol. Observations were carried out with a microscope equipped with the Cool SNAP-Pro Color Digital Camera (Olympus, Tokyo, Japan). The ImageJ program was used to perform the quantitative measurements of zebrafish larvae [34].

### 4.12. Statistical Analysis

Data were analyzed using the GraphPad Prism 7 statistical analysis package (version 5.01; GraphPad Software Inc., San Diego, CA, USA). All data were obtained in duplicate and represented as the mean ± standard deviation. The significance levels were evaluated using a one-way analysis of variance (ANOVA) and Dunnett’s multiple-range test.

## 5. Conclusions

This study provides insights into the anti-inflammatory potential of OFH–PH, which involves the regulation of inflammatory end products. In summary, this study strongly evidenced that OFH–PH exerts significant anti-inflammatory activity against LPS stimulation by regulating the production of inflammatory factors (NO, TNF-α, IL-6, and PGE2) in macrophages. The downregulation of the iNOS and COX-2 protein expressions further confirmed the inhibitory potential of NO and PGE2 production. Additionally, the protective effect of OFH–PH was evaluated against an LPS-induced zebrafish model. To the best of our knowledge, this is the first report on the anti-inflammatory activity of hydrolysates obtained from *P. olivaceus* byproducts. The obtained results indicate that OFH–PH has great potential as an anti-inflammatory candidate for the production of functional compounds. Additionally, OFH–PH may be beneficial in the medicinal and pharmaceutical sectors for the treatment of inflammatory disorders.

## Figures and Tables

**Table 1:** Proximate composition of dried flounder fish head.

Constituents	Proximate Composition (%)
Protein	56.48 ± 3.02
Lipid	17.01 ± 1.45
Polysaccharide	5.61 ± 0.68
Ash	17.51 ± 1.35
Moisture	3.39 ± 0.98

Values represent mean ± SD from triplicate determinations.

**Table 2:** Hydrolytic yields and the protein contents of enzymatic hydrolysates.

Hydrolysate	Yield (%)	Protein Content (%)
Control (Distilled water)	18.24 ± 0.22	34.55 ± 1.47
Alcalase	39.00 ± 2.94 ***	43.05 ± 1.23 **
Kojizyme	35.67 ± 0.47 **	47.62 ± 0.61 ****
Flavourzyme	36.33 ± 3.40 **	41.46 ± 1.76 **
Neutrase	39.33 ± 3.40 ***	43.81 ± 0.46 ***
Protamax	44.33 ± 3.77 ***	52.06 ± 0.15 ****
Pepsin	46.50 ± 0.50 ****	45.97 ± 0.77 ***
Trypsin	26.67 ± 1.25	40.56 ± 1.07 *

Values represent mean ± SD from triplicate determinations. Significance was compared relative to the control group at * *p* < 0.05, ** *p* < 0.01, *** *p* < 0.005, **** *p* < 0.0001.

**Table 3:** IC_50_ values of NO production of the enzymatic hydrolysates.

Hydrolysate	IC_50_ Value (µg/mL)
Alcalase	731.74 ± 8.47 ****
Kojizyme	902.93 ± 11.72 ****
Flavourzyme	451.72 ± 10.24 ***
Neutrase	1033.33 ± 6.28 ****
Protamax	1357.89 ± 14.94 ****
Pepsin	299.82 ± 4.18
Trypsin	1800.12 ± 19.55 ****

Values represent mean ± SD from duplicate determinations. *** *p* < 0.005, and **** *p* < 0.0001 compared to pepsin hydrolysate.

**Table 4:** Amino acid composition of OFH–PH.

Constituent Amino Acids	Composition mg/g	Percentage (%)
Aspartic acid	69.93	8.16
Threonine	34.03	3.97
Serine	42.02	4.90
Glutamic acid	110.50	12.89
Proline	72.97	8.51
Glycine	133.64	15.59
Alanine	73.54	8.58
Cysteine	3.18	0.37
Valine	33.73	3.94
Methionine	24.37	2.84
Isoleucine	23.37	2.73
Leucin	45.25	5.28
Tyrosine	16.42	1.92
Phenylalanine	27.06	3.16
Histadine	23.14	2.70
Lysine	52.71	6.15
Arginine	71.18	8.30
Total	857.09	100.00

## Data Availability

All data are contained within the manuscript.
